# A tritrophic signal that attracts parasitoids to host-damaged plants withstands disruption by non-host herbivores

**DOI:** 10.1186/1471-2229-10-247

**Published:** 2010-11-15

**Authors:** Matthias Erb, Nicolas Foresti, Ted CJ Turlings

**Affiliations:** 1 Laboratory for Fundamental and Applied Research in Chemical Ecology, Institute of Biology, University of Neuchâtel, Neuchâtel, Switzerland

## Abstract

**Background:**

Volatiles emitted by herbivore-infested plants are highly attractive to parasitoids and therefore have been proposed to be part of an indirect plant defense strategy. However, this proposed function of the plant-provided signals remains controversial, and it is unclear how specific and reliable the signals are under natural conditions with simultaneous feeding by multiple herbivores. Phloem feeders in particular are assumed to interfere with plant defense responses. Therefore, we investigated how attack by the piercing-sucking cicadellid *Euscelidius variegatus *influences signaling by maize plants in response to the chewing herbivore *Spodoptera littoralis*.

**Results:**

The parasitoid *Cotesia marginiventris *strongly preferred volatiles of plants infested with its host *S. littoralis*. Overall, the volatile emissions induced by *S. littoralis *and *E. variegatus *were similar, but higher levels of certain wound-released compounds may have allowed the wasps to specifically recognize plants infested by hosts. Expression levels of defense marker genes and further behavioral bioassays with the parasitoid showed that neither the physiological defense responses nor the attractiveness of *S. littoralis *infested plants were altered by simultaneous *E. variegatus *attack.

**Conclusions:**

Our findings imply that plant defense responses to herbivory can be more robust than generally assumed and that ensuing volatiles convey specific information about the type of herbivore that is attacking a plant, even in complex situations with multiple herbivores. Hence, the results of this study support the notion that herbivore-induced plant volatiles may be part of a plant's indirect defense stratagem.

## Background

Predators and parasitoids are important natural enemies of herbivorous insects. By reducing the abundance of herbivores, they can help protect plants from damage. Parasitoids in particular can use herbivore-induced plant volatiles (HIPVs) as host-searching cues [1,2]. Such volatile-mediated tritrophic interactions have a considerable potential to shape ecosystem dynamics [3], but it remains unclear to what extend the plant signals are emitted by the plant to specifically attract natural enemies of herbivores [4]. If HIPVs are indeed emitted by the plant to attract the third tropic level, specificity of the signals should be an important aspect of the interactions [5]. This is particularly relevant under natural conditions, where plants are often attacked by non-hosts or by multiple herbivores simultaneously. While specialist parasitoids can distinguish between plants attacked by hosts and plants attacked by non-hosts using HIPV cues [6], the impact of non-hosts feeding *on the same plant *as the host could be problematic. Of the few studies on this topic, most seem to suggest that non-hosts (or non-prey in the case of predators) may interfere with the plant's responses in several ways: They can indirectly change plant resistance [7-9] and therefore influence feeding behavior and subsequent HIPV induction of hosts, influence the induction of plant defenses on the physiological level via positive or negative cross-talk [10,11] or induce volatile bouquets that either mask [12] or distort [13,14] host/prey-finding cues. Yet, if HIPVs have evolved in the context of tritrophic interactions, it can be expected that in tightly co-evolved systems, parasitoids will be able to "tolerate" the effects of non-host herbivores on the plant and recognize volatiles indicating host presence, even in a complex situation with multiple herbivore attacking the same plants. Similarly, plants should have evolved to minimize the negative interference of herbivores with their defense responses targeted at other attackers. To date, little is known about this alleged robustness of volatile-mediated tritrophic interactions.

In the case of parasitoids, their capacity to learn to associate particular volatile blends with the presence of hosts may help them to cope with environmental complexity [15]: many parasitoids are more attracted to a specific volatile blend after exposure during host encounters [16]. This may also allow parasitoids to reduce the impact of the negative interference of non-host herbivores on host-induced volatiles [17].

Typical examples of negative interference between herbivores come from research comparing insects with different feeding styles. Chewing herbivores predominantly activate jasmonic acid (JA) and ethylene (ET) dependent defenses [18,19], whereas many piercing-sucking insects appear to induce defense-pathways commonly associated with pathogens [10,20,21]. The silverleaf whitefly *Bemisia tabaci *for example induces salicylic acid (SA)-dependent defenses and suppresses jasmonic acid (JA)-dependent plant reactions in *Arabidopsis thaliana *[22], possibly via classical JA/SA cross-talk [23,24]. In a particularly illustrative recent study, it was found that *B. tabaci *reduces the attractiveness of spider mite infested lima beans to predatory mites [25]. The effects of piercing-sucking insects on plant defense are also evident at the gene expression level: In *A. thaliana*, *B. tabaci *induces the SA-responsive genes *PR1 *and *PR5 *and reduces the expression of the JA markers *PDF1.2 *and *VSP *[22]. In lima bean, the expression of *LOX*, a key enzyme in JA-biosynthesis is repressed by *B. tabaci *alongside *PIOS*, the gene coding for the enzyme β-ocimene synthase [25]. The evident potential of piercing sucking insects to interfere with the responses of plant to other herbivores makes them important factors to consider in studies on the functionality of plant defenses in multitrophic systems.

This prompted us to conduct experiments on the impact of the piercing-sucking leafhopper *Euscelidius variegatus *(Hemiptera : Cicadellidae; Kirschbaum 1858) on volatile emissions of maize plants and the tritrophic interaction involving maize, the lepidopteran pest *Spodoptera littoralis *(Boisduval) (Lepidoptera: Noctuidae) and the generalist parasitoid *Cotesia marginiventris *(Cresson) (Hymenoptera: Braconidae). *C. marginiventris *is a solitary larval endoparasitoid that can use a broad range of noctuid moths, including many maize pests, as hosts. It is generally highly responsive to induced volatile cues provided by the host-damaged plants, including maize [16,26]. Leafhoppers like *E. variegatus *co-occur with chewing lepidopteran larvae in maize agroecosystems as well as in nature on teosinte, the wild ancestor of maize [27], making the system a logical candidate for the current study. Based on the results from earlier studies (see above), we hypothesized that *E. variegatus *affects the plant's response to *S. littoralis*, thereby interfering with the ability of *C. marginiventris *to locate its host with the use of HIPVs. Following this, we specifically tested if *C. marginiventris *is able to distinguish HIPVs from hosts (*E. variegatus*) and non-host (*S. littoralis*) and if simultaneous *E. variegatus *attack affects the attractiveness of *S. littoralis *infested plants. The assays presented here took into account the effect of previous encounters with hosts and host-associated odours, which can strongly affect the responses of parasitoids, including *C. marginiventris *[16,17], through associative learning [28].

## Methods

### Insects and insect treatments

The cicadellid *E. variegatus *was reared on 4-8 week old barley plants in plastic Bugdorm cages (Megaview, Taiwan) under standardized conditions (25°C, 16:8 h L/D). The caterpillar *S. littoralis *(Boisduval) (Lepidoptera: Noctuidae) and the solitary endoparasitoid *C. marginiventris *(Cresson) (Hymenoptera: Braconidae) were reared as previously described [29]. Adult parasitoids were kept in plastic cages at a male/female ratio of approximately 1:2 and were provided with moist cotton wool and honey as food source. Cages were kept in incubators (25°C; 16:8 h L/D) and transferred to the bioassay laboratory 30 min before the experiments. Two to four day old mated naive and experienced females were tested. For details on the training setup see D'Alessandro *et al*., [30]. Naïve wasps did not have any previous oviposition experience and had never before been in contact with plant odours. To experience wasps, they were either placed with the host (20 L2 *S. littoralis *larvae) until they had oviposited 3-5 times, or with the non-host (10 adult *E. variegatus*) during 2 minutes, while they were exposed to the odor from plants infested by *E. variegatus *and/or *S. littoralis*. This resulted in 5 different experience groups (Table 1): Host contact (*S. littoralis*) in presence of *S. littoralis *induced volatiles (SS), *E. variegatus *induced volatiles (SE) and double-infestation induced volatiles (SES) as well as non-host contact (*E. variegatus*) in the presence of *S. littoralis *induced volatiles (ES) and *E. variegatus *induced volatiles (EE). As the perception of odors in the absence of herbivorous insects does not change the responsiveness of *C. marginiventris *[17], this treatment was not included. The different groups of wasps were kept separately in small plastic boxes and released into the olfactometer 1-3 h after their experience.

**Table 1:** Herbivores and odor blends used to train *C. marginiventris*.

Experience	Contact (Insect)	Odor Blend (Plant)
**Naïve**	-	-
**SS**	*S. littoralis *(host)	*S. littoralis *induced
**SE**	*S. littoralis *(host)	*E. variegatus *induced
**SES**	*S. littoralis *(host)	*E. variegatus *and *S. littoralis *induced
**ES**	*E. variegatus *(non-host)	*S. littoralis *induced
**EE**	*E. variegatus *(non-host)	*E. variegatus *induced

Naive *C. marginiventris *females were exposed to either host or non-host insects (column 2) in the presence of different plant odour blends (column 3). This resulted in differently experienced wasps (column 1).

### Plants and odor sources

Maize (*Zea mays*, var. Delprim) was sown in plastic pots (10 cm high, 4 cm diameter) with commercial potting soil (Ricoter Aussaaterde, Aarberg, Switzerland) and placed in a climate chamber (23°C, 60% r.h., 16:8 h L/D, 50'000 lm/m^2^). Plants used for the experiments were 10-12 d old and had 2-3 fully developed leaves. The evening before the experiments, plants were transferred to glass vessels [29] and infested with second instar *S. littoralis *larvae (released in the whorl of the youngest leaf) or *E. variegatus *adults (released freely into the vessel). For the experiment involving double-infestation, maize seedlings were inoculated with *E. variegatus *adults 48 hours before the olfactometer assay and second instar *S. littoralis *larvae were added the evening before the experiment.

Thirty *E. variegatus *adults per plant were used, a density that induced a reliable response of maize seedlings without impairing their physiology: Preliminary experiments had shown that infestation with 20 hoppers/plant resulted in a relatively weak and less consistent volatile response (data not shown), whereas densities much above 30 individuals per plant led to a phenomenon called "hopper burn", where the plant's vascular architecture is so severely impaired that the plants start displaying yellow discoloration (personal observations).

For the chewing herbivore, nine *S. littoralis *larvae where used in the double-infestation experiment (for both the double and the single herbivore treatments), while the density was reduced to three in the single infestation experiment (comparing *S. littoralis *with *E. variegatus *infested plants). This lower density of *S. littoralis *was chosen because it resulted in a similar response in the maize plants as infestation by thirty *E. variegatus *(see results), allowing for a better qualitative comparison of the plant's defensive response. After infestation, the vessels with the plants and herbivores were attached to the air supply of the olfactometer and kept under laboratory conditions (25°C, 50% r.h., 16:8 h L/D, humidified airflow 0.3 l/min, 8000 lm/m2). Olfactometer experiments were done the following day, between 10 A.M. and 4 P.M.

### Olfactometer bioassays

The different odor sources were tested for attractiveness to parasitoids in a six-arm olfactometer as described in Turlings *et al*., [29]. To compare the attractiveness of plants infested with one of the two herbivores, we compared 4 sources: *E. variegatus *infested plants, *S. littoralis *infested plants, uninfested plants and clean air. To only test the attractiveness of *E. variegatus *infested plants, they were tested against uninfested plants. In the double-infestation experiment, attraction to *S. littoralis *infested plants was compared to plants infested with both herbivores and uninfested plants. In every experiment, one arm per odor source was used. The remaining arms were connected to empty vessels and carried clean, humidified air only. The position of the odor sources was randomly assigned for each experimental run to avoid position-bias. Purified and humidified air entered each odor source vessel at 1.2 l/min (adjusted by a manifold with six flowmeters, Analytical Research System, Gainesville, FL, USA) via Teflon tubing and carried the volatiles through to the olfactometer compartment. Half of the air (0.6 l/min/olfactometer arm) was pulled out via volatile collection traps that were attached to a port on top of each odor source vessel (see "Collection and analyses of HIPVs"). These traps, as well as the wasp release chamber were connected to a vacuum pump via Tygon tubing and flow meters, and airflows were balanced with a pressure gauge. Wasps were released in groups of six into the central part of the olfactometer, alternating between groups of naive and experienced wasps, and after 30 min the wasps that had entered an arm of the olfactometer were counted and removed. Wasps that did not enter an arm after this time were removed from the central part of the olfactometer and considered as "no choice." Each experiment was performed 6-8 times on different days. This resulted in 6-8 independent replicates for each olfactometer setup.

### Collection and analysis of HIPVs

HIPVs of each odor source were collected during the olfactometer bioassay on a Super-Q trap (25 mg, 80-100 mesh; Alltech Associates, Deerfield, IL, USA, described by Heath & Manukian, [31]. Each trap was attached horizontally to the elbow of an odor source vessel and connected via Tygon tubing to a flowmeter (Analytical Research System) and a vacuum pump. Air carrying the volatiles was pulled through each trap at a rate of 0.6 l/min during each behavioral bioassay. Afterwards, the traps were extracted with 150 μl dichloromethane (Suprasolv; Merck, Dietikon, Switzerland), and 200 ng of n-octane and n-nonyl acetate (Sigma, Buchs, Switzerland) in 10 μl dichloromethane were added to the samples as internal standards. All extracts were stored at -76°C until analyses. Traps were washed with 3 ml dichloromethane before they were reused for a next collection. HIPVs of the experiments were identified with a gas chromatograph (Agilent 6890 Series GC system G1530A) coupled to a mass spectrometer that operated in electron impact mode (Agilent 5973 Network Mass Selective Detector; transfer line 230°C, source 230°C, ionization potential 70 eV, scan range 33-280 amu). A 2-μl aliquot of each sample was injected in the pulsed splitless mode onto an apolar capillary column (HP-1, 30 m, 0.25 mm ID, 0.25 μm film thickness; Alltech Associates). Helium at constant flow (0.9 ml/min) was used as carrier gas. After injection, the column temperature was maintained at 40°C for 3 min and then increased to 100°C at 8°C/min and subsequently to 200°C at 5°C/min followed by a postrun of 5 min at 250°C. The detected volatiles were identified by comparison of their mass spectra with those of the NIST 05 library, by comparison of their spectra and retention times with those of authentic standards, and by comparison of retention times with those from previous analyses [32]. Precise quantification of the identified volatiles was carried out using an Agilent 6850 gas chromatograph with a flame ionization detector. A 3-μl aliquot of each sample was injected in pulsed splitless mode onto the same type of column as above at a constant pressure of 18.55 psi. The column temperature ramping was as described above.

### Analysis of gene expression

The leaves from plants used in the olfactometer experiments were harvested and flash-frozen in liquid nitrogen. Based on the volatile profiles, 5 representative samples of each treatment were chosen and ground to a fine power under liquid nitrogen. Total RNA was then extracted using Quiagen RNA-Easy extraction kits following the manufacturer's instructions. The quality of the RNA was assessed by photometry and gel electrophoresis. To remove contaminant genomic DNA, all samples were treated with Ambion DNAse following the manufacturer's protocol. cDNA was then synthesized using Invitrogen Super-Script III reverse transcriptase according to the manufacturer's instructions. Based on current knowledge about the molecular basis of maize responses to insect attack, we used *Zm-B73LOX *[33], *Zm-AOC *and *Zm-AOS *[34] as markers for the induction of the octadecanoid pathway, *Zm-SerPIN *[33], *Zm-MPI *and *Zm-Bx1 *[35] as markers for the induction of direct defenses and *Zm-HPL *[36], *Zm-TPS10 *[37], *Zm-TPS23 *[38] and *Zm-IGL *[39] as markers for volatile induction. Quantitative reverse transcriptase real time polymerase chain reactions (q-PCR) where carried out using the following gene-specific primers: *Zm-AOS *L:acctgttcacgggcacctac; R:cgaggagcgaggagaagttg. *Zm-AOC *L: ccccttcaccaacaaggtgt; R: accgagatgtggccgtagtc. *Zm-B73LOX *L: gcgacaccatgaccatcaac; R: gctcggtgaagttccagctc. *Zm-SerPIN *L: gacggaggaggaaggaggag; R: acctgatgcactgcttgcac. *Zm-MPI *L: atgagctccacggagtgc; R: acctgatgcactgcttgcac. *Zm-BX1 *L: cccgagcacgtaaagcagat; R: cttcatgcccctggcatact. *Zm-HPL *L: acttcggcttcaccatcctg; R: gtagtagcccggccagatga; Zm-IGL L: gcctcatagttcccgacctc; R: gaatcctcgtgaagctcgtg. *Zm-TPS10 *L: tgtgtccacggtccaatgtt; R: gtccgctgtccttgcaaaat. *Zm-TPS23 *L: tctggatgatgggagtcttctttg; R: gcgttgccttcctctgtgg. The q-PCR mix consisted of 5 ul Quantace Sensimix containing Sybr Green I, 3.4 ul H20, 100 nmol of each primer (2 × 0.3 ul H20) and 1 ul of cDNA sample. Q-PCR was carried out using 45 cycles with the following temperature curve: 10s 95°C, 20s 60°, 15s 72°. The final melt curve was obtained by ramping from 68 to 98°C in 1°C steps every 5s. To determine primer efficiencies and optimal quantification thresholds, a dilution series of a cDNA mix consisting of 4 ul solution from every sample was created. Six 10-fold dilution steps were carried out to determine primer efficiencies and the standard curve was included into every q-PCR run. The final obtained Ct values (using the automated threshold determination feature of the Rotor-Gene 6000 software) were corrected for the housekeeping gene GapC and normalized to control levels to obtain average fold changes of treated plants.

### Herbivore performance

To test for a possible effect of *E. variegatus *infestation on the growth and feeding activity of *S. littoralis*, the double-infestation setup (see above) was used. Nine second instar *S. littoralis *larvae were weighed before the experiment and were then put on maize seedlings that had either been infested with *E. variegatus *or had initially been left herbivore free. After the olfactometer tests (20 h, see above), the larvae were removed from the plants and weighed again to determine their change in body mass. This procedure was carried out for 5 olfactometer runs (see above), resulting in 5 replicate values for caterpillar growth per treatment. Our previous studies on this system have shown that resistance against *S. littoralis *can reliably be quantified with feeding-bioassays ranging from 6-24 h [8,33,40].

### Statistical analysis

The relationship between parasitoids' behavioral responses and the different odor sources offered in the six-arm olfactometer was examined with a log-linear model (a generalized linear model, GLM). As the data did not conform to simple variance assumptions implied in using the multinomial distribution, we used quasi-likelihood functions to compensate for the overdispersion of wasps within the olfactometer [29]. The model was fitted by maximum quasi-likelihood estimation in the software package R (R: A language and Environment for Statistical Computing, Version 1.9.1, Vienna, Austria, 2006, ISBN 3-900051-07-0 http://www.R-project.org), and its adequacy was assessed through likelihood ratio statistics and examination of residuals. The amounts of volatiles and gene expression data were analyzed by using ANOVAs followed by Holm-Sidak post-hoc tests. Datasets that were not normally distributed were transformed prior to analysis. Where transformation did not resolve non-normality or unequal variances, ANOVA's on ranks followed by Dunn's or Student-Newman-Keul's post-hoc tests were used.

## Results

### Parasitoid attraction

**Figure 1:**
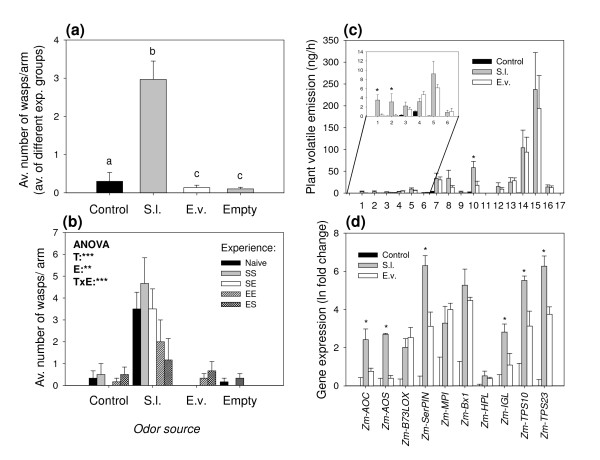
**Influence of *E. variegatus *and *S. littoralis *infestation on parasitoid attraction, volatile emission and defense gene expression**. **(a)**: Choice of *C. marginiventris*, shown as the average numbers (+SE) of wasps per release (groups of 6) and olfactometer arm. Different experience types are pooled. Control = Uninfested plant; S.l. = *S. littoralis *infested plant; E.v. = *E. variegatus *infested plant; Empty = Empty arms. Different letters indicate significant differences between treatments (p < 0.05) (n = 6). **(b)**: Average choice of *C. marginiventris *parasitoids with different previous host- or non-host experience. Naive = No experience; SS = Host-presence with *S. littoralis *induced plant odours; SE = Host presence with *E. variegatus *induced plant odours. EE = Non-host presence with *E. variegatus *induced plant odours; ES = Non-host presence with *S. littoralis *induced odours. Stars denote significant effects of treatment (T), experience (E) and the interaction (TxE) (*p < 0.05, **p < 0.01, ***p < 0.001). **(c)**: Average volatile emission (+SE) of herbivore infested maize seedlings. 1 = (Z)-3-hexenal; 2 = (E)-2-hexenal; 3 = (Z)-3-hexen-1-ol; 4 = β-myrcene; 5 = (Z)-3-hexenyl acetate; 6 = (Z)-β-ocimene^N^; 7 = linalool; 8 = (3E)-4,8-dimethyl-1,3,7-nonatriene (DMNT); 9 = phenethyl acetate; 10 = indole; 11 = methyl anthranilate; 12 = geranyl acetate; 13 = E- β-caryophyllene; 14 = (E)-α-bergamotene; 15 = E-β-farnesene; 16 = β-sesquiphellandrene^N ^17 = unknown sesquiterpene^N^. Compounds denoted with "N" were only tentatively identified. Stars denote significant differences between *S. littoralis *and *E. variegatus *induced plants (p < 0.05). **(d)**: Average change in gene expression (+SE) of herbivore infested maize seedlings relative to uninfested control plants. Stars in graphs **(c) **and **(d) **denote significant differences between *S. littoralis *and *E. variegatus *induced plants (p < 0.05).

**Figure 2:**
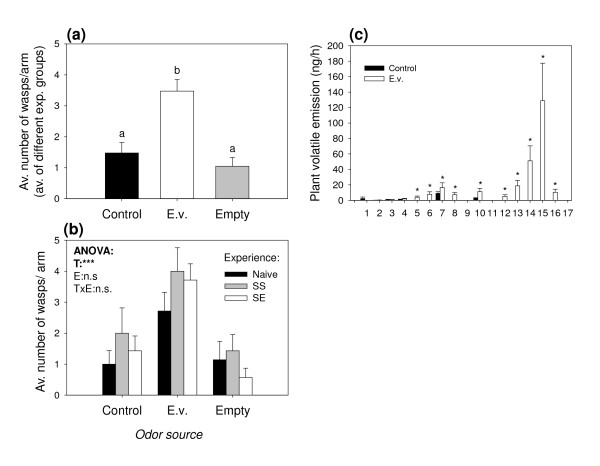
**Influence of *E. variegatus *on parasitoid attraction and plant volatile emission**. **(a)**: Choice of *C. marginiventris*, shown as the average numbers (+SE) of wasps per release (groups of 6) and olfactometer arm. Control = Uninfested plant; E.v. = *E. variegatus *infested plant; Empty = Empty arms. Different letters indicate significant differences between treatments (p < 0.05). **(b)**: Average choice of *C. marginiventris *parasitoids with different previous host-experience. Naive = No experience; SS = Host-presence with *S. littoralis *induced plant odours; SE = Host presence with *E. variegatus *induced plant odours. Stars denote significant effects of treatment (T), experience (E) and the interaction (TxE) (*p < 0.05, **p < 0.01, ***p < 0.001). **(c)**: Average volatile emission (+SE) of *E. variegatus *infested maize seedlings. For compound descriptions, see legend in Figure 1. Stars denote significant differences between *E. variegatus *induced and uninfested plants (p < 0.05). (n = 7)

**Figure 3:**
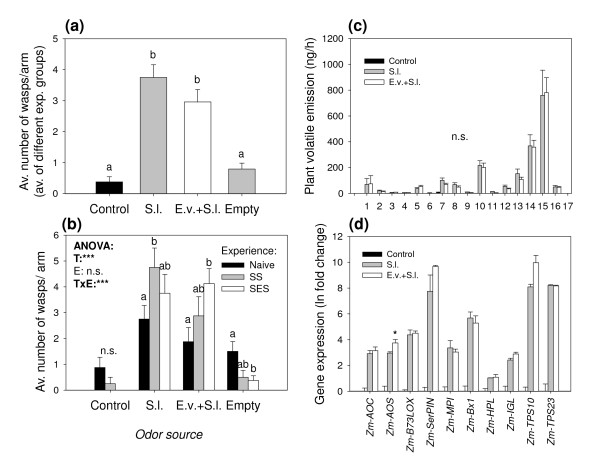
**Influence of *E. variegatus *on *S. littoralis *induced parasitoid attraction, volatile emission and defense gene expression**. **(a)**: Choice of *C. marginiventris*, shown as the average numbers (+SE) of wasps per release (groups of 6) and olfactometer arm. Control = Uninfested plant; S.l. = *S. littoralis *infested plant; E.v.+S.l. = *E. variegatus *and *S. littoralis *infested plant; Empty = Empty arms. Different letters indicate significant differences between treatments (p < 0.05). (n = 8). **(b)**: Average choice of *C. marginiventris *parasitoids with different previous host-experience. Naive = No experience; SS = Host-presence with *S. littoralis *induced plant odours; SES = Host presence with *E. variegatus *and *S. littoralis *induced plant odours. Different letters denote a significant difference between experience type within a treatment (p < 0.05). Stars denote significant effects of treatment (T), experience (E) and the interaction (TxE) (*p < 0.05, **p < 0.01, ***p < 0.001). **(c)**: Average volatile emission (+SE) of herbivore infested maize seedlings. For compound descriptions, see legend in Figure 1. **(d)**: Average change in gene expression (+SE) of herbivore infested maize seedlings relative to uninfested control plants. Stars denote significant differences between *S. littoralis *and *E. variegatus *and *S. littoralis *induced plants (p < 0.05) (n = 3).

**Figure 4:**
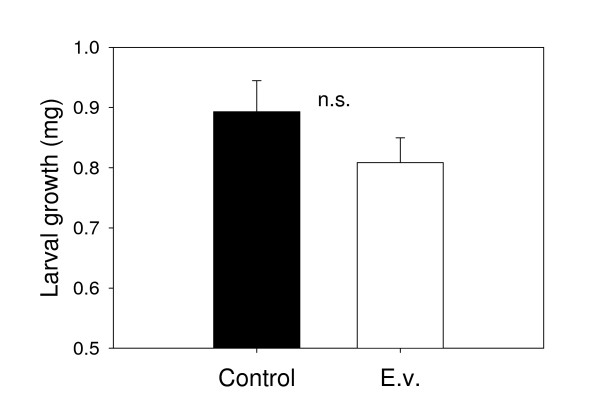
**Influence of *E. variegatus *on maize resistance to *S. littoralis***. Average *S. littoralis *growth (+SE) over 18 hours on uninfested (Control) and *E. variegatus *infested (E.v.) plants (n = 5).

To test whether the parasitoid *C. marginiventris *is able to distinguish between volatile blends from plants infested with its host *S. littoralis *and volatiles emitted by plants infested with the non-host *E. variegatus*, we performed an olfactometer assay giving the wasps a choice between uninfested, *S. littoralis*-, and *E. variegatus*-infested plants. Irrespective of their previous experience, *C. marginiventris *exhibited a strong preference for odors from host-infested plants (Figure 1a and 1b). *E. variegatus*-infested plants were even less attractive than control plants. Interestingly, previous contact with *E. variegatus *over a period of 2 minutes drastically reduced the overall responsiveness of the wasps and the choice for the odor of host-infested plants: Compared to naïve and host-experienced wasps, wasp choice was reduced by 50% after they had contacted *E. variegatus *while being exposed to the odor of *E. variegatus *infested plants and by two thirds after they encountered *E. variegatus *in association with the odor of *S. littoralis*-infested plants (Figure 1b). This apparent effect of negative association was reflected in a significant effect of the type of experience and the interaction term (treatment*experience) in the GLM. Because *C. marginiventris *seemed to prefer control plants over *E. variegatus*-infested plants in the first olfactometer assay, we tested if the *E. variegatus *infestation had a repellent effect on the parasitoid. When offered an uninfested plant and an *E. variegatus *infested plant only, *C. marginiventris *showed a preference for odors from *E. variegatus*-infested plants (Figure 2a), irrespective of previous experience (Figure 2b). To assess whether *E. variegatus *attack affected the plants' response to *S. littoralis*, we infested the plants with *E. variegatus *and *S. littoralis *simultaneously or with *S. littoralis *only. Overall, *C. marginiventris *was equally attracted to double infested plants compared to plants infested with *S. littoralis *only (Figure 3a). However, parasitoids with previous positive experience, while perceiving the odor of double infested plants, shifted their preference in favor of this odor (Figure 3b). *S. littoralis *gained similar amounts of weight irrespective of the presence of *E. variegatus *(Figure 4), indicating similar feeding activity.

### Volatile profiles

The volatile profiles analyzed from the olfactometer experiments described above show that plants infested with 30 *E. variegatus *adults emitted similar amounts of terpenoids as seedlings infested with 3 *S. littoralis *caterpillars (Figure 1c). Therefore, the overall induction resulting from the two chosen herbivore densities was comparable. Unlike *S. littoralis*, *E. variegatus *feeding did not result in detectable release of the green leaf volatiles (GLVs) (*Z*)-3-hexanal and (*E*)-2 hexenal, whereas the emission of the other two GLVs, (*Z*)-3-hexen-1-ol and (*E*)-2-hexenyl acetate was not significantly different between the two herbivore treatments. Of the other compounds, only indole was emitted in lower amounts by *E. variegatus*-infested plants compared to *S. littoralis*-infested plants (Figure 1c). The analyzed volatile profiles of the second olfactometer experiment (*E. variegatus *vs. control) confirmed that *E. variegatus *induces the same 18 compounds commonly found in *S. littoralis*-infested plants, apart from the two GLVs (*Z*)-3-hexanal and (*E*)-2-hexenal (Figure 2c). The volatile compounds induced by simultaneous *E. variegatus *and *S. littoralis *attack were qualitatively and quantitatively similar to the profile induced by *S. littoralis *only (Figure 3c). Compounds that were not identified by comparing retention times and spectra with those of pure standards are indicated in Figures 1 and 2 with superscript N, and their identity should be considered tentative.

### Gene expression profiles

To gain insight into the genetic basis for the observed volatile responses, we profiled gene expression patterns of plants infested by *E. variegatus*, *S. littoralis *and both herbivores simultaneously. The putative octadecanoid biosynthesis genes were induced by both *E. variegatus *and *S. littoralis*, with *Zm-AOS *showing a reaction to *S. littoralis *only and *Zm-AOC *being more strongly induced by the caterpillar (Figure 1d). The markers for direct defenses, *Zm-SerPIN, Zm-MPI*, two proteinase inhibitor genes, and *Zm-BX1*, a key gene for the synthesis of hydroxamic acids, also responded to both herbivores, with *Zm-SerPIN *showing a stronger reaction to *S. littoralis*. Three genes implicated in volatile production (*Zm-IGL*, the indole synthase, *Zm-TPS10 *and *Zm-TPS23*, two major terpenoid synthases) showed a similar pattern (Figure 1d). *Zm-HPL*, a gene typically involved in GLV synthesis, was only significantly induced by *S. littoralis*, but there was no significant difference compared to the transcriptional activity upon *E. variegatus *attack. Plants attacked simultaneously by both *E. variegatus *and *S. littoralis *showed comparable levels of expression for all genes under investigation (Figure 3d), with the exception of *Zm-AOS*, which showed a more pronounced response upon double attack compared to *S. littoralis *infestation only.

## Discussion

### Differences in induced volatile and defense gene profiles

Surprisingly, *E. variegatus *induced volatile profiles resembled the ones induced by *S. littoralis *in many aspects. Both herbivores induced a variety of mono- homo- and sesquiterpenes, the shikimic acid pathway derived indole, as well as volatile metabolites from the oxlipin cascade, (*Z*)-3-hexen-1-ol and (*E*)-2-hexenyl acetate (Figures 1c and 2c). This suggests that the plant's volatile response to the two herbivores is not fundamentally different, although the induction by *E. variegatus *was much weaker on a *per capita *basis. Sesquiterpene emissions in maize are strongly correlated with induction of JA, resulting from activation of the octadecanoid pathway [41]. Furthermore, the transcriptional profiling shows an induction of genes involved in JA biosynthesis (*Zm-B73LOX *and *Zm-AOC*, Figure 1d) by *E. variegatus*. Our results therefore imply that *E. variegatus *induces JA-dependent volatile production rather than suppress this type of defense, as has been shown for other piercing-sucking insects [20,22].

The fact that *E. variegatus *did not measurably alter *S. littoralis*-induced volatile emissions (Figure 3c) and did not reduce the induced resistance the caterpillars encountered in the leaves (Figure 4) is further evidence for the absence of negative cross-talk between defense pathways in this system, as it has been reported in other cases [22,25]. This notion is confirmed by the transcriptional data, showing similar induction of most defense-related genes upon *S. littoralis *attack irrespective of the presence of *E. variegatus *(Figure 3d). Only the expression of *Zm-AOS*, a putative maize allene oxide synthase involved in OPDA synthesis [34], was slightly higher in the double treatment than after caterpillar attack alone. It remains to be investigated if this higher expression leads to any changes in octadecanoid pathway dynamics.

### Specific attraction of *C. marginiventris*

*C. marginiventris *showed a clear preference for *S. littoralis*-induced blend of volatiles (Figure 1a), suggesting that the parasitoid can readily distinguish between the odors induced by the two herbivores. Both the main sesquiterpenes and aromatic compounds emitted by insect-damaged maize plants are known to be of minor importance for innate attraction of *C. marginiventris *females [30,32,37], while fresh damage has been shown to be highly attractive [42]. These types of volatiles were clearly more prominent in *S. littoralis*-infested plants, which exhibited physical tissue damage and consequently released more (*Z*)-3-hexanal and (*E*)-2-hexenal (Figure 1c). We thus provide evidence for the notion that the key attractants for this parasitoid are likely to be found in the blend directly released from wounded sites. The fact that *C. marginiventris *was attracted to *E. variegatus *infested plants in the absence of host-induced volatiles (Figure 2a) demonstrates that there is no repellent effect of *E. variegatus *induced volatiles *per se*. This could mean that the key attractants responsible for the attractiveness of the *S. littoralis*-induced blend were also emitted by *E. variegatus*-infested plants, albeit in much lower quantities, prompting the insect to respond to them only in the absence of stronger cues. Alternatively, *C. marginiventris *may simply choose to follow "the most promising trail" that is present in an environment by using secondary cues in the absence of primary attractants. This behavioral plasticity could be especially important for generalist parasitoids, as they have to be able to exploit a broad range of host-induced cues [28].

The attractiveness of *S. littoralis*-infested plants was not reduced when *E. variegatus *was present on the same plants (Figure 3a), indicating a robust host-finding behavior of the parasitoid. This contrasts with other studies documenting effects of multiple herbivory on tritrophic systems. Rodriguez-Sanoa *et al*. [43] found that *C. marginiventris *was more attracted to tomato plants infested with both the aphid *Macrosiphum euphorbiae *and the caterpillar host *Spodoptera exigua *than plants infested with *S. exigua *only. Similarly, the green peach aphid *Myzus persicae *increased the attractiveness of spider-mite induced pepper plants to a generalist predator [44]. Zhang *et al*. [25]on the other hand show that *B. tabaci *infestation reduces the attractiveness of spider mite induced plant to predatory mites. As shown in the same study, the interference of phloem feeders with host plant physiology can be density dependent, and it is possible that this is one of the reasons for the varying results in the literature. In maize agroecosystems, as well as in populations of teosinte, the wild ancestor of maize in Mexico, leafhoppers densities do not exceed 15 individuals per plant [45,46]. Early instar *Spodoptera spp*. larvae can initially occur at densities of up to 100 individuals per plant (G. von Merey, unpublished). Thus, the herbivore densities used in our experiment (30 *E. variegatus *adults, 3- 9 *S. littoralis *larvae) were strongly biased to maximize the chance of finding a possible effect of the leafhoppers on the volatile signals. The fact that even under these conditions, the tritrophic interaction between maize, *S. littoralis *and *C. marginiventris *remained fully functional is strong evidence for its robustness. The *C. marginiventris *strain that was used in this study originates from Mexico, where there is a long co-evolutionary history of the parasitoid, *Spodoptera *spp. and the ancestors of maize. This may be another explanation for the observed robustness of the system, which is unlikely to be found in the more artificial combinations of plants and insects that have been studied in this context.

It will be interesting to assess if *C. marginiventris *is also able to distinguish host-induced volatile blends from those induced by chewing non-host herbivores. In maize, early instars of non-host stem-borers like *Ostrinia numbilalis *and *Diatrea *spp. inflict visually similar leaf-damage as *Spodoptera spp*., and further studies should determine if *C. marginiventris *has adapted to avoid possible mismatches at this level as well. As yet, no other study on the subject matter has taken into account the learning ability of parasitoids and predators, which can be, as shown here, an important factor affecting their foraging behavior.

### Associative learning by the parasitoid

Parasitoids commonly show an ability to learn to associate specific olfactory or visual cues with the presence of hosts and food [47-50]. This associative learning is thought to help the parasitoids to optimize their foraging success by focusing on the most rewarding cues [2,28]. The results obtained here confirm this notion and moreover represent one of the rare examples of negative learning, whereby non-rewarding cues become less attractive [51-53]. *C. marginiventris *markedly decreased its response to induced maize volatiles after having perceived them during contact with *E. variegatus *adults for two minutes (Figure 1b). While such effects have been demonstrated for host-free habitats [51,52] and in the context of previously learned responses [53], we show here -to our knowledge for the first time- that the presence of non-hosts can have direct negative effects on the responsiveness of previously inexperienced parasitoids. Similar assays have shown that the responsiveness of *C. marginiventris *is not affected when it perceives odors in the absence of herbivores [17], suggesting that it is indeed the presence of a non-host rather than the absence of hosts that led to the observed reduction in choice fidelity. It is tempting to speculate that *C. marginiventris *may have adapted to specifically recognize non-hosts like leafhoppers, which it regularly encounters in nature [27].

The attractiveness of *S. littoralis *damaged plants was not reduced by positive experience with alternative volatile blends (Figure 1b), implying that there is a strong innate preference of *C. marginiventris *for the compounds resulting from fresh damage. This could be adaptive, as it enables the parasitoid to discriminate between plants attacked by chewing herbivores (potential hosts) and insects with other feeding modes (non-hosts). On the other hand, positive associative learning enabled the parasitoid to distinguish double- from single-infested plants after oviposition-experience in presence of the respective blends (Figure 3b). Rasmann and Turlings [54] found something similar when they tested the attraction of *C. marginiventris *to maize plants that were simultaneously attacked by *S. littoralis *and a belowground herbivore. In both cases the learned behavior cannot be explained by the measurable volatile profiles, as they did not differ between the two treatments (Figure 3c; [54]). This confirms the importance of minor, undetected compounds, which can be affected by the presence of the additional herbivore and that can be learned and thus affect the wasps responses. As concluded in earlier studies [32,54], future research will have to focus on these elusive signals in order to unravel the functional complexity of herbivore-induced volatiles in detail.

## Conclusions

Our data show that plant-mediated signaling in the tritrophic system comprising maize, the lepidopteran pest *S. littoralis*, and the parasitoid *C. marginiventris *is not disrupted by a non-host phloem feeder. This demonstrates that this specific interaction, unlike some others, is robust and that the attraction of natural enemies of herbivores to plant signals can also function when plants are attacked by multiple antagonists. Yet, flexibility in the use of reliable cues is maintained through associative learning, which may help parasitoids to specifically focus on the odor of plants that carry potential hosts and avoid plants that are only attacked by non-hosts. These results support the still controversial notion that HIPVs, at least in part, serve as functional signals to attract the enemies of the enemies of plants [1,3,5,55].

## Authors' contributions

ME designed the experiments, analyzed the data and wrote the manuscript. NF contributed to the experimental design, carried out the experiments and analyzed data. TCJT conceived of the study, participated in its design and helped to draft the manuscript. All authors have read and approved the final manuscript.
